# Landslide Susceptibility Evaluation Using Different Slope Units Based on BP Neural Network

**DOI:** 10.1155/2022/9923775

**Published:** 2022-05-23

**Authors:** Jianling Huang, Xiaoye Zeng, Lu Ding, Yang Yin, Yange Li

**Affiliations:** ^1^Department of Engineering Management, School of Civil Engineering, Central South University, Changsha, Hunan 410083, China; ^2^Pricing Certificate Centre, Changde Municipal Development and Reform Commission, Changde, Hunan 415000, China

## Abstract

Landslides are one of the most widespread natural hazards that cause damage to both property and life every year. Therefore, the landslide susceptibility evaluation is necessary for land hazard assessment and mitigation of landslide-related losses. Selecting an appropriate mapping unit is an essential step for landslide susceptibility evaluation. This study tested the back propagation (BP) neural network technique to develop a landslide susceptibility map in Qingchuan County, Sichuan Province, China. It compared the results of applying six different slope unit scales for landslide susceptibility maps obtained using hydrological analysis. We prepared a dataset comprising 973 historical landslide locations and six conditioning factors (elevation, slope degree, aspect, lithology, distance to fault lines, and distance to drainage network) to construct a geospatial database and divided the data into the training and testing datasets. We based on the BP learning algorithm to generate landslide susceptibility maps using the training dataset. We divided Qingchuan County into six different scales of slope unit: 4,401, 13,146, 39,251, 46,504, 56,570, and 69,013, then calculated the receiver operating characteristic (ROC) curve, and used the area under the curve (AUC) for the quantitative evaluation of 6 different slope unit scales of landslide susceptibility maps using the testing dataset. The verification results indicated that the evaluation generated by 56,570 slope units had the highest accuracy with a ROC curve of 0.9424. Overelaborate and rough division of slope units may not get the best evaluation results, and it is necessary to obtain the slope units most consistent with the actual situation through debugging. The results of this study will be useful for the development of landslide hazard mitigation strategies.

## 1. Introduction

Landslides are among the most important natural disasters that cause extensive losses worldwide in human life and property [1, 2]. In China, it is reported that 6,181 geo-hazards occurred in 2019 (of which 68.2% were landslides), resulting in 299 people injured or dead and a direct economic loss of 2.77 billion CNY (https://www.stats.gov.cn/tjsj/ndsj/2020/indexch.htm). How to prevent landslide-related disasters actively and effectively and develop regional disaster prevention and early warning measures is one of the main issues around the world [3]. It is necessary to identify the landslide areas to prevent landslide-related disasters, and landslide susceptibility assessment is a primary tool for solving the problem [4]. Therefore, assessing models related to landslide susceptibility has become a principal research topic worldwide in recent years [5].

Landslide susceptibility reflects the variation of landslide occurrences in a given area based on local geo-environmental factors and thus represents where landslides are likely to occur [6, 7]. Landslide susceptibility mapping (LSM) is the first and most important step in landslide susceptibility assessment [1]. The construction of landslide susceptibility maps is essential to understand and predict future landslides and mitigate the consequences of landslides in the study area [8]. The extraction and delineation of map units are a key element affecting the accuracy of the zoning and the reliability of the results, which is also the basis for landslide hazard analysis [9]. In earlier studies, scholars have proposed various methods to delineate land landscapes for landslide susceptibility assessment [10]. In general, the map units commonly used for landslide susceptibility analysis are grid units and slope units [11]. A grid unit is a mapping unit in which the area is divided into regular squares of predetermined dimensions [12]. Many scholars have carried out a major number of landslide susceptibility researchers based on this unit type [13, 14]. However, although the units are simple to segment, the topographic boundaries are not regular quadrilaterals in nature. Thus, the method does not reflect the characteristics of the actual topography. It is based solely on size, without considering the constraints of the topographic boundaries, making it easy for the units to cross the topographic line [15]. Therefore, the slope unit was proposed. It approximates the entire study area consisting of several slopes of different sizes and is an approximate description of the actual slope boundary [16]. The results of a large number of researches have shown that slope units, compared with grid units, can preserve the integrity of slopes; improve the degree of conformity with the actual topography; represent the actual development of landslide hazards in the region better; and thus enhance the accuracy and efficiency of spatial prediction of landslides [17, 18]. The most commonly used method of slope unit delineation is the hydrological analysis model [19, 20]. It is now widely accepted and has been integrated into the toolbox of Spatial Analyst Tools—Hydrology in ArcGIS. However, the classification of slope units by hydrological analysis lacks unified standards. Few studies have focused on the influence of slope units of different classification scales on the evaluation results of landslide susceptibility.

Over the past three decades, advancements in geographic information systems (GIS) and remote sensing (RS) technologies, as well as the development and application of methods and techniques to assess landslide susceptibility, hazards, and risks, have proven to be feasible and effective [21–24]. The methods using GIS and SR allow a more accurate landslide susceptibility assessment than previous approaches [25, 26]. RS-derived data and GIS spatial analysis tools form the basis of LSM [27]. In general, the applied methods could be classified into four types: a probabilistic analysis based on landslide cataloging, qualitative analysis based on empirical reasoning, deterministic modeling methods, and semi-quantitative methods for applying mathematical and statistical models. As a major basic technical tool, the landslide cataloging method has been used by various scholars in landslide analysis and assessment in the early stage [28–30]. A qualitative analysis method is a risk assessment of the study area based on expert experience or relevant knowledge to classify the risk level. This method is mainly used for early landslide susceptibility evaluation and is more subjective [31]. The deterministic modeling method is based on mechanisms and processes that control the deformation and failure of landslides, which requires extremely detailed parameters of spatial variables, and it is applicable only at large scales over small areas [32–34]. A semi-quantitative method is a mathematical model represented by regression analysis, discriminant analysis, and other methods. It is based on mathematical statistics and explores the objective law of developing things from a nonlinear perspective, with solid persuasive power and practical value [35, 36]. In recent years, various machine learning algorithms such as artificial neural networks [37, 38], random forest [39, 40], maximum entropy [41], and naive Bayes [42, 43] have been successfully used in a wide range of applications and be optimal for data handling.

Currently, landslide susceptibility evaluation based on slope units has been widely used. In contrast, few studies have focused on the influence of slope units of different division scales on the results of landslide susceptibility evaluation. Therefore, it is important to explore and study the influence of slope units with different division scales on landslide susceptibility evaluation results to find out the accurate division method, which is of theoretical significance to improve the accuracy of landslide susceptibility evaluation. We address the shortage in the literature by investigating a hybrid integration approach of GIS and back propagation neural network for landslide susceptibility evaluation, which is based on different scale slope units with a case study at the Qingchuan County in China. By comparing and analyzing the results of landslide susceptibility mapping in Qingchuan County based on different division scale slope units, additionally, the results have been evaluated using the receiver operating characteristic curve (ROC) and the area under the curve (AUC). This study makes up for the deficiency of single scale in the study of susceptibility mapping, provides technical support and reference for disaster prevention and mitigation in Qingchuan County, and also provides a useful reference for future landslide disaster prevention and control.

## 2. General Situation of the Study Area

**Figure 1:**
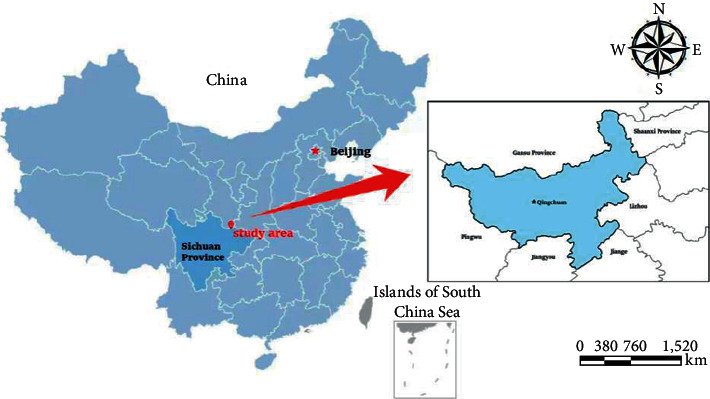
Location map of the study area.

The study area of Qingchuan County occupies an area of 3,216 km^2^ in the Sichuan Province of China (Figure 1). Qingchuan County is located on the northern edge of Sichuan Basin, which suffered a great deal of damage following landslide since the Wenchuan earthquake, and was selected as a suitable site for the evaluation of landslide susceptibility model. It extends from 104°36′E and 105°38′E and from 32°12′N and 32°56′N. Elevations range from 491 m to 3837 m. It is surrounded by steep mountains with a cut area of 500–1,500 m, and the slope ≥25 accounts for 76.8%. All strata are found in this area except for the Cretaceous, Jurassic, and Ordovician, which are missing. Magmatic, metamorphic, and clastic rocks are the most widely distributed. The soft and hard lithologies alternate due to the old and new tectonic movements. There are two major fault zones in this area: one is the Qiaozhuang fault in the north and the other is the Chaba fault in the south. Both of them have a NE trend and run through the whole area. The study area is located within a subtropical monsoon climate regime, the annual average temperature is 13.7°C, and the average annual rainfall is 932.9 mm. The average amount of annual sunshine is 1,238 h, with a relative sunshine duration of 30%.

**Figure 2:**
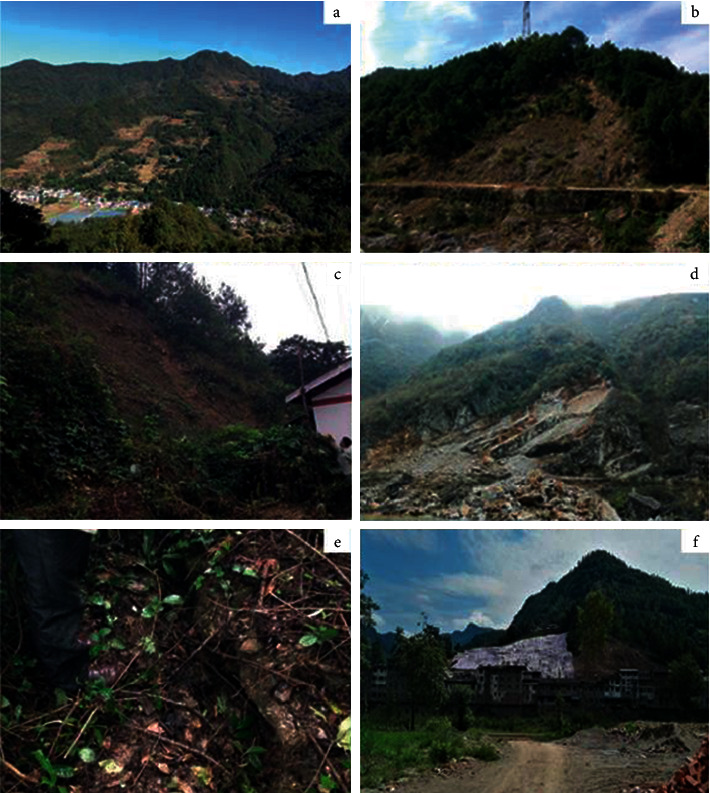
(a) Full view of an old-style resurrection-type landslide in Qingchuan County. (b) Shallow loose accumulation landslide in the side of a river. (c) Shallow loose accumulation landslide occurred behind the house of residents. (d) Consequent bedding rockslide near a village. (e) Landslide caused by the Wenchuan earthquake. (f) Landslide caused by artificial slope cutting of a new road.

Due to the difference in seismicity meteorological conditions (such as rainfall) and human engineering in the topographic and geotechnical structural fault zone, landslide disasters in the study area are different. According to the analysis of landslide disaster data in this area, the causes of regional landslides can be summarized as the follows: (a) old-style resurrection-type landslide (Figure 2(a)): in natural conditions, the old landslide is in a stable or basically stable state with fertile soil and stable water in the mountainous area, an important habitat for humanity suitable for residents to live and carry out agricultural activities. Human activities have a great influence on the stability of the old landslide. Meanwhile, there are two fault zones in Qingchuan County, seismicity is high frequency, and fracture structure is abundant. Influenced by the earthquake, rainfall, and human engineering activities, it is easy to cause the whole or partial landslide to resurrect, resulting in the movement or destruction of the slope, thus developing into a landslide disaster. (b) Shallow loose accumulation landslide (Figures 2(b) and 2(c)): due to the influence of rainfall, weathering, and surface inclination, the surface layer is vulnerable to damage and deformation, which slides along the bedrock cover discontinuity, thus forming a landslide disaster. (c) Consequent bedding rockslide (Figure 2(d)): the main types of rocks in the area are metamorphic rocks, mainly phyllite and shale—the bedrock is controlled by soft structural planes such as gentle dip cleavage and bedding, and dip slope. Due to earthquakes and weathering, joints and cracks are formed in the upper part, and the rock mass structure is broken. With the influence of human engineering activities and rainfall, rock bottoms slide along weak structural surfaces such as cleavage and bedding, resulting in landslides. (d) Earthquake caused landslide (Figure 2(e)): Qingchuan County is an important disaster area affected by the Wenchuan earthquake. Under the influence of seismic load, the stability was greatly reduced, and the deformation and damage were intense. Tens of thousands of tensile cracks were generated on the top of the slope, thus forming the potentially unstable landslide. (e) Man-made slope cutting caused landslide (Figure 2(f)): after the Wenchuan earthquake, Qingchuan County carried out post-disaster reconstruction, large-scale housing construction, and repair or expansion of the existing road leading to the serious slope cutting phenomenon. Some slopes have a thin overburden, which causes the upper part of the slope to be hollow after slope cutting, thus reducing the slip resistance force of the soil. The landslide was caused by tilting and deformation of slopes and the soil-rock interface under gravity and rainfall.

**Figure 3:**
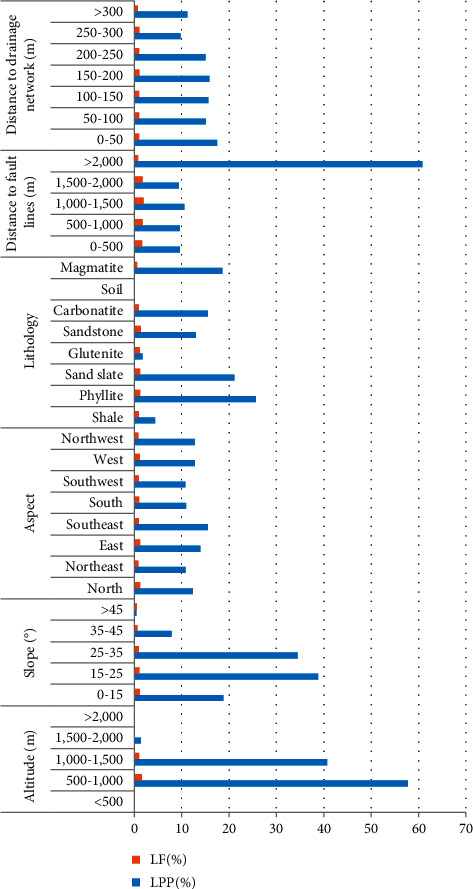
LF and LPP for the landslide predisposing factor classes.

To compare the number and area of landslide, the percentage of landslide point (LPP) and the frequency of landslide (LF) are used to represent the activity degree of landslide in different factors. LPP is expressed as the ratio in percentage between the number of landslides and the total landslides in each predisposing factor class. LF is described as the ratio in percentage between LPP and the rate of slope units (SUP). LPP, LF, and SUP are calculated using the following formulas:

(1) $$\begin{array}{c} LPP=\frac{H}{M_{h}},\\ \text{SUP}=\frac{V}{M_{v}},\\ LF=\frac{LPP}{\text{SUP}}, \end{array}$$

where *H* indicates the number of landslides in each interval, *M*_*h*_ indicates the total landslides, *V* stands for the number of slope units in each interval, and *M*_*v*_ represents the total slope units. The statistical results are shown in Figure 3. At the same time, the LF of different factors in different intervals will also be used as the quantized values to eliminate the differences between dimensions in the later model evaluation.

**Figure 4:**
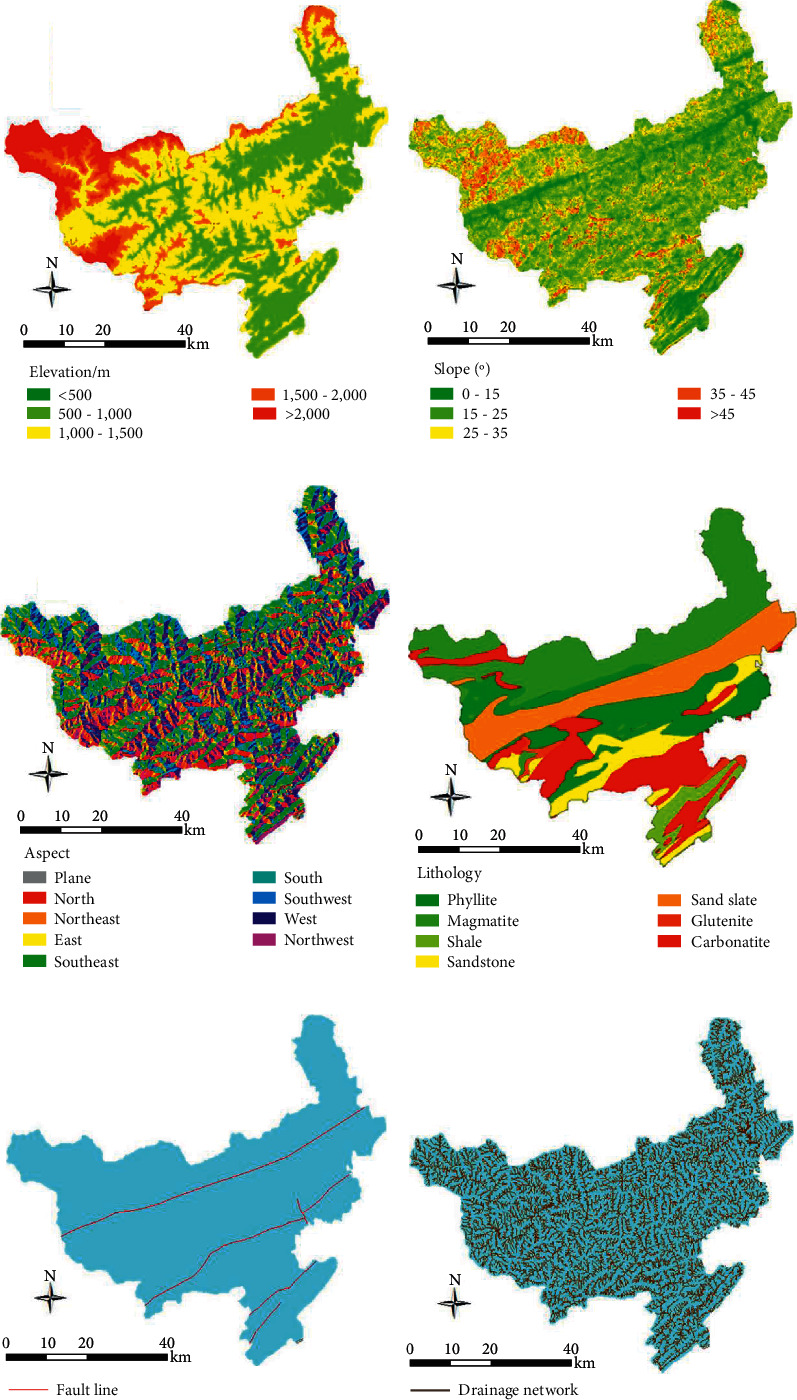
Landslide predisposing factor maps used for the landslide susceptibility analysis: (a) elevation; (b) slope; (c) aspect; (d) lithology; (e) distance to faults lines; and (f) distance to drainage network.

The elevation is a parameter frequently used in landslide susceptibility analysis and is regarded as a vital factor for susceptibility mapping [44–46]. Taking the equal interval of 500 m, the elevation values of the study area were classified into five categories: ＜500, 500–1,000, 1000–1,500, 1,500–2,000, and ＞2,000 (Figure 4(a)). It can be seen in combination with Figure 3, almost all landslides are in the range of 500–1,500, and the LF values in this range are greater than or extremely close to 1. It shows that the elevation interval has an essential influence on the occurrence of landslides. Starting from 1,500 m, LPP and LF of landslide values decrease with the increase in elevation. Severe weathering causes the slope to slow down at the high elevations, and the slope body tends to stabilize. At low elevations, the slope is steep, combined with river erosion and human activity, causing landslides frequently.

The slope was considered the main parameter influencing slope stability [47] and is widely used in landslide susceptibility analyses [38, 48]. The entire slope in this study was divided into five categories: <15°, 15–25°, 25–35°, 35–45°, and >45° (Figure 4(b)). It can be seen that the maximum distribution of landslides was observed in the classes 15–25° (38.78%) and 25–35° (34.44%). The LF values greater than 1 or extremely close to 1 were in the range of <15°, 15–25°, and 25–35°, which indicated that the three ranges were most conducive to the development of landslide collapse.

The aspect was considered an important factor in landslide susceptibility [14, 18]. Aspect areas were classified into eight classes (Figure 4(c)). The spatial relationship between aspect and landslide is presented in Figure 3. It shows that the maximum distribution of landslides was observed in the area with the east (13.97%) and southeast (15.54%). That is, landslides occurred in sunny slops more than in shady slopes. Under the same conditions, sunny slop is full of sunlight, rainfall, and soil moisture, all of which lead to the critical state of landslide initiation that is lower than on shady slope [49–51].

Lithology was also considered the main factor influencing slope stability [51]. The variability of lithologies cropping out in this study area allows grouping into seven lithologic classes (Figure 4(d)). The relationship between the lithology and landslide was analyzed (Figure 3), the result shows that landslides occurred predominately on phyllite, sericite silt slate, and magmatite, respectively, of 25.63%, 21.1%, and 18.59%. In addition, the values of LP for most lithologies were over 1 or close to 1. The maximum value was obtained for the sericite silt slate.

There are two main fault lines throughout the study area. One of them is divided into two branches within the area (Figure 4(e)). To assess cause-effect relationships between fault lines and landslide, distances to faults were calculated using five intervals: 0–500 m, 500–1,000 m, 1,000–1,500 m, 1,500–2,000 m, and >2,000 m (Figure 3). The statistical result shows that the maximum distribution of landslides was observed in the classes >2,000 m (60.86%).

The drainage system plays a vital role in the development of landslides [48, 50]. To evaluate the degree to which the drainage network influence the occurrence of landslide, the distance to drainage network was calculated using buffers with the following seven intervals: 0–50 m, 50–100 m, 100–150 m, 150–200 m, 200–250 m, 250–300 m, and >300 m. The LPP of each buffer is shown in Figure 3, and the landslide points were distributed uniformly, but in terms of the values of LP, landslides were more likely to occur within 300 m from drainage.

## 3. Methodology

### 3.1. Division of Slope Units

**Figure 5:**
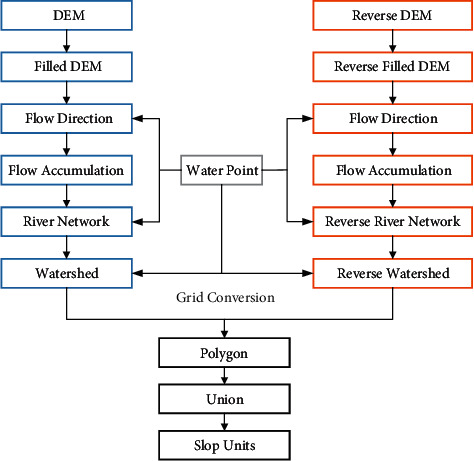
Flow chart of slop unit division method based on hydrological analysis.

An optimal slope unit subdivision for landslide susceptibility evaluation cannot be determined unequivocally, and the quality and precision of landslide susceptibility modeling rely on the subdivision of slope units [7, 9, 52]. Partitioning slope units reasonably could improve the precision of landslide susceptibility zonation [53]. The commonly used hydrological analysis model, based on ridgelines and valley lines, divides watersheds into separate slopes [9, 54]. The framework to generate slope units is illustrated in Figure 5.

### 3.2. BP Neural Network Model

Many methods are available for landslide susceptibility evaluation, and in summary, two major types of methods are available, that is, deterministic models and nondeterministic models. Deterministic models mainly use the traditional calculation model of landslide damage mechanics and basic spatial data to predict landslide hazard susceptibility, since the deterministic model must collect a large amount of data about the specific topography, hydrogeology, and other aspects of the slope. Even though it can explain the mechanics of slope damage, however, it is only suitable for assessing the landslide susceptibility of a single entity, but not for studying regional landslides. A nondeterministic model based on statistical analysis theory is established to evaluate the landslide susceptibility of the study area by superimposing factors affecting slope stability according to the weights. Unlike the deterministic model, the nondeterministic model does not require data related to the physical characteristics of landslides, but analyses the relationship between historical landslides and landslide influencing factors and uses statistical methods to predict the likelihood of future landslides. In contrast, it is better to use nondeterministic models for the evaluation and prediction of landslide susceptibility on a large scale.

A BP neural network is a multistage training feedforward network based on an error back propagation algorithm, which is also one of the most widely used neural network models. The BP neural network is a parallel distributed processing method that does not need to determine the input-output pattern mapping relationship beforehand, but stores and adaptively learns the output value similar to the desired result. Owing to its strong modeling ability and fault tolerance, a BP neural network has a great research value in the field of landslide geological hazard susceptibility evaluation.

#### 3.2.1. Data Processing

To reduce the error of the BP neural network, it is necessary to normalize the input data before using the landslide susceptibility evaluation model. Assume that the input and output data contain qualitative data. In that case, the qualitative data must be converted to quantitative data before normalization to avoid the situation where the network is unable to be identified. Among the 6 evaluation indicators selected in this study, there are qualitative and quantitative factors. The qualitative or quantitative classification of the factors is shown in Table 1. As far as quantitative factors are concerned, each has a different dimension. When dividing landslide susceptibility areas, superimposing two or more attributes that have no connection in meaning or concept, such as slope and lithology, will only produce meaningless results. Only by transforming the meaning of the attributes of the data layers and placing qualitative and quantitative factors of different scales on a platform of the same scale they can be substituted into the relevant models for landslide hazard susceptibility assessment. In this study, the landslide frequency of each indicator is used as the quantitative value of the indicator to achieve the quantification of the indicator and the uniformity of the scale.

The “frequency of slippage” data for each evaluation indicator were quantified between [0, 1] using the Z score standardization method.

(2) $$\begin{array}{c} S=\frac{x-\mu }{\sigma }, \end{array}$$

where *S* means quantized value, *x* means the value of landslide frequency, *μ* stands for the mean value of the landslide frequency of the indicator, and *σ* represents the standard deviation of the landslide frequency of the indicator. The quantified data are shown in Table 2.

#### 3.2.2. Modeling

The BP neural network is very computationally intensive if calculated manually, and this study is operated with the help of SPSS Clementine software. 39251 slope units are randomly arranged and divided into two categories: training samples and test samples, of which 80% of the training samples are 31,400 and 20% of the test samples are 7,851.

First of all, the input, output, and hidden layers of the BP neural network should be determined. The input layer is the quantified value of LF for the six predisposing factors mentioned above in 31,400 slope units. The output layer is 0 or 1. 0 means no landslide has occurred, and 1 means landslide has occurred. The hidden layer has been repeatedly tested, adjusted, and compared. Finally, the number of nodes in the implicit layer of the network is determined to be 3. The following 10 data from the training sample are selected for illustration, as shown in Table 3.

After setting up the input and output layer data and the hidden layer, the expected error and learning rate must be determined to stop network training when the target is reached. Due to the inconsistency between the network generalization ability and the network expectation error, more minor network errors require more hidden nodes and more training time. All other things being equal, the network target error was set to five different levels of 0.1, 0.01, 0.005, 0.001, and 0.0001. The training data with target errors of 0.005, 0.001, and 0.0001 were fitted with high accuracy by comparing the study. The expected and actual outputs of the training data were not significantly inaccurate. However, during the test, the error between the expected and actual outputs of the test data was significant, reflecting the fact that these 3 are networks with poor generalization capabilities and cannot be used. When training the network with a set target error of 0.1 was performed, a faster network fit occurred. However, at the same time, there were significant errors. When comparing the desired output with the actual output for both the training and test data, both have significant errors, reflecting poor prediction accuracy, and therefore cannot be used. Finally, experimentation and comparison determined that an expected error of 0.01 was more appropriate.

In addition, the learning rate plays an important role in BP neural networks as it affects the amount of variation in the connection weight coefficients produced in each network cycle. Too high a learning rate can make the system unstable, while too low a rate can make the network converge slower and take longer to train. For general training, the tendency is to start with a small learning rate to maintain the stability of the system and then gradually increase the value to a value appropriate for the network model. Based on previous experience, the learning rate was taken to be in the range of [0.01, 0.8]. The learning rate of the BP neural network model in this study was determined to be 0.01 using trial algorithms for comparison.

The training function is a function that comes with the software. The expected error is set to 0.01, the learning rate is 0.01, the initial weights are randomly selected between 0.1 and 0.3, and the number of training sessions is 10,000 times. The parameters are set and brought into the software for operation. The trained model was also tested against 7851 test samples after training, and the test results showed that the training model was feasible.

After training and testing, the influencing factor data of 39,251 samples were brought into the previously trained BP network model to output the predicted values between [0, 1], where a value closer to 1 represents a higher probability of a landslide occurring in that slope unit and closer to 0 represents a lower probability of a landslide occurring in that slope unit. Again, 10 sets of data were brought from the prediction sample for illustration, as shown in Table 4.

#### 3.2.3. Weight Analysis of Influencing Factors

**Figure 6:**
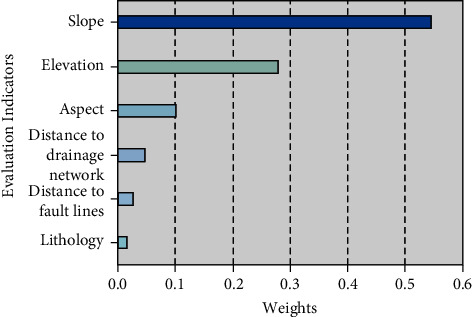
Weight of each evaluation index.

Due to the self-learning feature of the BP neural network, there is no need to determine the weights of influencing factors in advance, which avoids the error of subjective determination of weights. The neural network can get the most appropriate and objective weights of influencing factors through actual data and continuous learning training and then apply them to the subsequent model prediction. As shown in Figure 6, by constructing the BP neural network model and training and predicting the data, the weight values of each factor are 0.55 for slope, 0.28 for elevation, 0.11 for slope direction, 0.04 for distance from drainage network, 0.02 for distance from fault lines, and 0.01 for lithology, respectively. It indicates that in Qingchuan County, the slope has a more significant influence on landslide hazard development, followed by elevation and slope direction. Distance to drainage network, distance to fault lines, and lithology have less effect.

## 4. Results and Discussion

### 4.1. Landslide Susceptibility Mapping through BP Neural Network Model

When dividing slope units, catchment area thresholds need to be entered manually in the process of generating catchment basins, and they directly determine the morphology of the generated digital river network. Catchment areas with larger thresholds will extract a sparser network and vice versa will extract a denser network. It also indirectly determines the number of segmented units. If the drainage network is sparse, fewer slope units will be divided; more slope units will be divided if the drainage network is dense. It can be found that the catchment area threshold has a significant influence on the formation and density of the drainage network and also indirectly on the effectiveness and accuracy of the traditional hydrological methods for extracting slope units.

**Figure 7:**
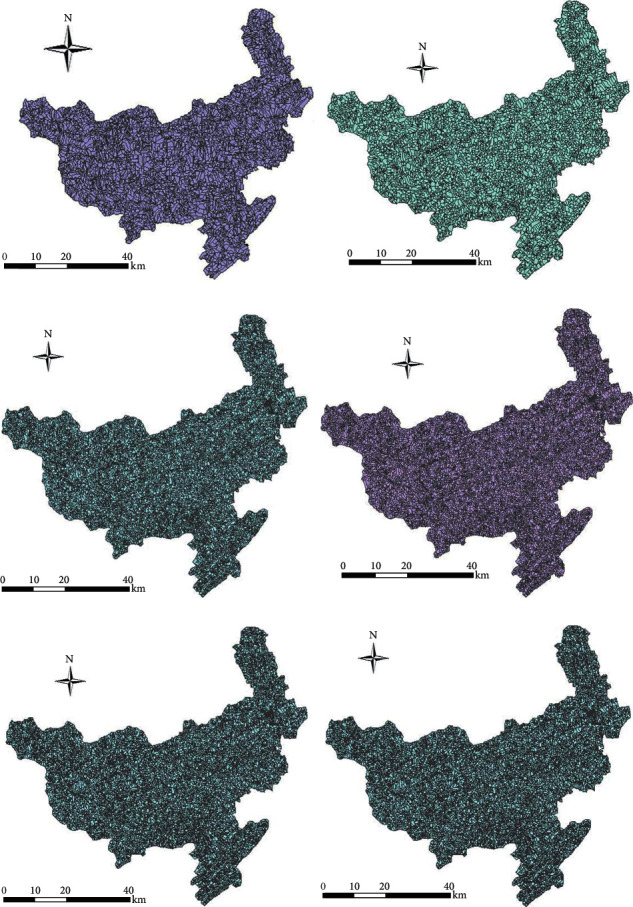
Slope units of different scales in Qingchuan County.

In response to the above problems, this study has additionally divided 4,401, 13,146, 39,251, 46,504, 56,570, and 69,013 slope units of different scales by dynamically adjusting the catchment area thresholds shown in Figure 7. After the slope units are divided, the six influencing factors of elevation, slope, slope direction, lithology, distance to fault lines, and distance to drainage network were studied and analyzed. A BP neural network model evaluates the susceptibility of landslide.

**Figure 8:**
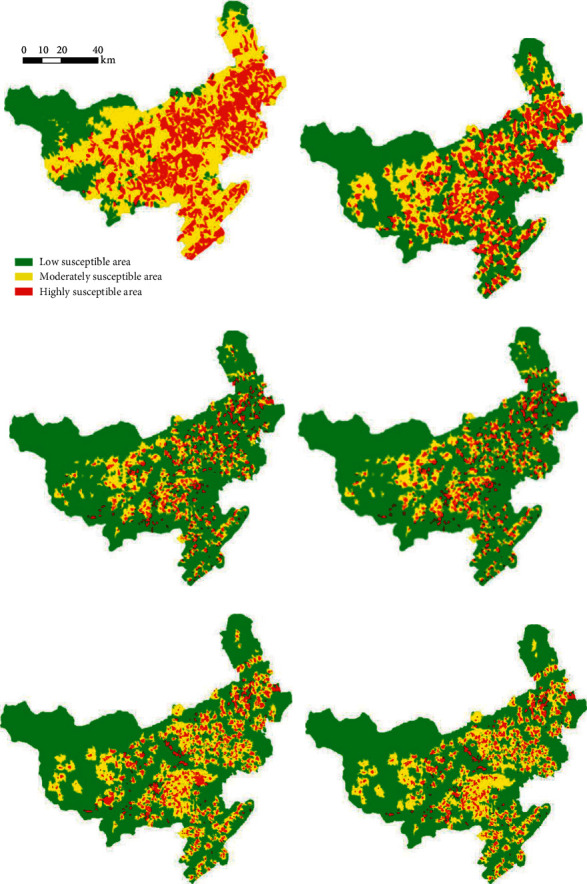
Landslide evaluation map of Qingchuan County for different scales of slope units.

The landslide susceptibility index obtained from the abovementioned BP neural network needs to be converted into a landslide susceptibility map to better manage landslide hazards. The susceptibility values of each unit were converted to raster format files in GIS to generate the final landslide susceptibility map. The pixel values were classified into three susceptibility classes: low, moderate, and high. The final six vulnerability evaluation partition maps were obtained according to the different scales of the divided slope units, as shown in Figure 8.

**Figure 9:**
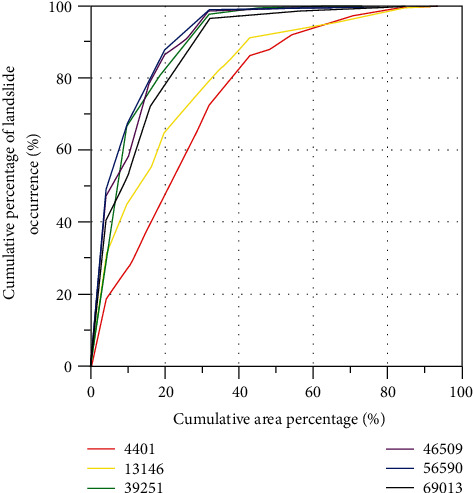
ROC curves of the landslide susceptibility model obtained using BP neural networks for different scale slope units.

It can be seen that the landslide susceptibility evaluation was generated by slope units classified according to different scales differs. The resulting landslide susceptibility model was then validated to verify the degree to which the model fitted/predicted landslide occurrence; this validation was achieved through a confusion matrix and ROC curves presented in Figure 9. The area under the curve (AUC) is one of the most effective indicators of a model's predictive accuracy. AUC generally ranges from 0.5 to 1.0. In the state of AUC >0.5, the closer the AUC is 1, the higher the accuracy is reflected. AUC is less accurate in the state of 0.5 to 0.7, while it responds to some accuracy in the case of 0.7 to 0.9 and has a higher accuracy above 0.9. The results of the validation are shown in Figure 9.

### 4.2. Validation of Landslide Susceptibility Maps

**Figure 10:**
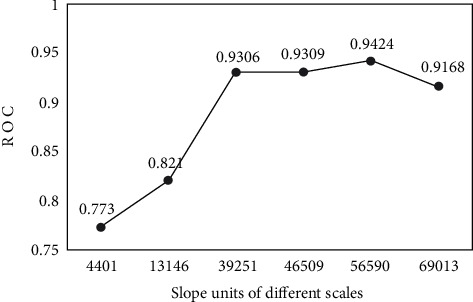
Accuracy trends of different scale slope units.

By verification and comparative analysis of the results of landslide hazard susceptibility analysis based on different scale slope units, the results are shown in Figure 10.

The AUC values for slope units 4,401, 13,146, 39,251, 46,509, 56,570, and 69,013 were 0.773, 0.821, 0.9306, 0.9309, 0.9424, and 0.9168, respectively. By analyzing Figures 9 and 10, it can be concluded that the value of AUC for each slope unit is higher than 0.77, which proves the good accuracy of the results based on six scale slope units selected in this study. Further analysis shows that the AUC value shows an increasing trend from 4,401 slope units to 56,570 slope units, with the smallest AUC value (0.773) at 4,401 and the maximum AUC value (0.9424) at 56,570. As the number of slope units continues to increase, the AUC value shows a decreasing trend from 0.9424 (56,570 slope units) to 0.9168 (69,013 slope units), indicating that the accuracy of the model evaluation will decrease when the number of slope units is greater than 56,570. In summary, from the ROC curve test results, the results for 39,251, 46,504, 56,570, and 69,013 slope cells are more accurate, while the results for 4,401 and 13,146 slope cells are less accurate compared with the other four results.

The division of slope units into overelaborate or too rough a degree of elaboration is not conducive to the evaluation of landslide susceptibility. 69,013 slope units are finely divided but do not give results of higher accuracy. In particular, it should be noted that the historical landslide data used to evaluate landslide susceptibility are generally point data. If the slope units are divided too finely, there is a possibility that the information from the historical landslide data will be segmented. The evaluation is negatively affected by replacing the actual information about the landslide with segmented local information about the landslide. In contrast, 4,401 slope units are rougher cells, which do not accurately represent factors such as aspect and slope, which are sensitive to unit size, and thus will also affect the accuracy of the evaluation results. In this study, 69,013 slope units are too delicate, while 4,401 and 13,146 slope units are rough, so the accuracy of the evaluation results of these three is worse than that of 39,251, 46,504, and 56,570.

Particularly, it is suggested that the 56,579 slope units are the most appropriate scale compared with the other five slope units in this study. In contrast, more accurate and appropriate map units may exist in practice.

At the same time, the division scale chosen in this study may not be optimal for different study areas, depending on the specific details of the study area and the historical landslide occurrence. At the same time, the division scale selected in this study may not be the best for the other areas, which depends on the specific details of the study area and the historical landslide occurrence.

### 4.3. Discussion

Landslide susceptibility evaluation is one of the important tools for landslide disaster prevention and mitigation. The suitability of map cell selection is directly related to the accuracy of landslide susceptibility mapping. Although the traditional grid cell-based partitioning method has a simple operation, it is not easy to present the spatial correlation between landforms, and it does not reflect the features of landforms, which often generates interference information in the partitioning results and affects the results and accuracy of classification. The slope unit is the basic unit for the development of geological hazards such as landslide and collapse, which is closely related to the geological environment conditions and can synthesize the effect of various control or influence factors. Most of the existing slope unit delineation methods use the principle of delineation based on catchment overlap; however, the accuracy of delineation results is limited by the determination of the catchment area threshold, with a high degree of subjective dependence and lack of uniform standards.

For the problem that the accuracy of the slope unit classification results is limited by the determination of the catchment area threshold and the high degree of subjective dependence, this study divides Qingchuan County into 4,401, 13,146, 46,504, 56,570, and 69,013 slope units and discusses the influence of different scales of slope units on the evaluation results. Using the ROC curve analysis and comparing the six zoning results based on different scales of slope units and BP neural network model, it can be seen that the scale of slope units will affect the accuracy of the results, and too fine or coarse slope units are not good for the results, so it is necessary to consider the trial algorithm to find the slope units that best match the actual situation.

## 5. Conclusions

This study selected six different scales of slope units, and a BP neural network model was computed to evaluate landslide susceptibility in Qingchuan County, a landslide-prone area in Sichuan Province, China. The study focuses on the following three issues: (1) selection of the factors influencing the evaluation of landslide susceptibility and using the Z score method to eliminate the differences between the magnitudes of the factors; (2) analysis of the spatial distribution characteristics of landslide geological hazards in the study area; and (3) evaluation and comparison of division results from slope units at different scales using the ROC and AUC. The result indicates that the evaluation generated by 56,570 slope units has the highest accuracy with a ROC curve of 0.9424. Overelaborate and rough division of slope units may not get the best evaluation results. Thus, it is necessary to obtain the most suitable slope unit for the actual situation through debugging.

Therefore, the landslide susceptibility map of Qingchuan County shows the areas prone to landslides and provides an informative map that can be used for the infrastructural planning process and land use. In addition, developing accurate landslide susceptibility maps can generate baseline information for further evaluation of landslide and related risk.

Finally, an important limitation lies in the fact is the determination of catchment thresholds. In the process of applying the hydrological analysis method, the catchment threshold determination is mainly through continuous debugging to select the most realistic segmentation threshold, which is computationally intensive. In the future, integration with advanced technologies in other fields will be enhanced to find more automatic and accurate segmentation methods.

## Figures and Tables

**Table 1:** Qualitative or quantitative classification of influencing factors.

Qualitative factors	Quantitative factors
Lithology	Elevation, slope, slope direction, distance to fault lines, distance to drainage network

**Table 2:** Landslide predisposing factors and their quantitative values.

Evaluation index	Class	Landslides		Slope units		LF (%)	Quantitative value
		Count	%	Count	%		
Elevation (m)	<500	0	0	1	0	0	0
	500–1,000	564	57.98	14,279	36.38	1.59	0.59
	1,000–1,500	395	40.65	16,124	41.08	0.99	0.37
	1,500–2,000	1	1.37	5,352	13.64	0.1	0.04
	>2,000	0	0	3,495	8.9	0	0

Slope (^○^)	0–15	182	18.76	6,209	15.82	1.18	0.27
	15–25	377	38.78	13,552	34.53	1.12	0.25
	25–35	335	34.44	14,672	37.38	0.92	0.21
	35–45	76	7.87	4,462	11.37	0.69	0.16
	>45	3	0.45	356	0.91	0.49	0.11

Slope direction	Plane	1	0.06	60	0.15	0.4	0.05
	North	119	12.33	3,916	9.98	1.24	0.14
	Northeast	105	10.82	4,906	12.5	0.87	0.1
	East	135	13.97	4,552	11.6	1.2	0.14
	Southeast	151	15.54	6,615	16.85	0.92	0.11
	South	106	10.95	4,313	10.99	1	0.12
	Southwest	104	10.76	4,661	11.87	0.91	0.11
	West	124	12.78	4,384	11.17	1.14	0.13
	Northwest	124	12.78	5,844	14.89	0.86	0.1

Lithology	Mudstone	41	4.43	1,845	4.7	0.94	0.12
	Millstone	249	25.63	8,003	20.39	1.27	0.17
	Sand slate	205	21.1	6,810	17.35	1.22	0.16
	Sand conglomerate	16	1.73	580	1.48	1.17	0.16
	Sandstone	126	13.01	3,688	9.4	1.38	0.18
	Carbonatite	150	15.51	6,562	16.72	0.93	0.12
	Magma	180	18.59	11,763	29.97	0.62	0.09

Distance to fault lines (m)	0–500	93	9.58	2,268	5.78	1.66	0.21
	500–1,000	93	9.58	2,120	5.4	1.77	0.22
	1,000–1,500	99	10.58	2,126	5.42	1.95	0.25
	1,500–2,000	88	9.4	2,099	5.35	1.76	0.22
	>2,000	592	60.86	30,638	78.06	0.78	0.1

Distance to drainage network (m)	0–50	170	17.48	6,688	17.04	1.03	0.15
	50–100	146	15.07	5,767	14.69	1.03	0.15
	100–150	151	15.62	6,054	15.42	1.01	0.14
	150–200	154	15.84	5,709	14.54	1.09	0.15
	200–250	146	15.01	5,697	14.51	1.03	0.15
	250–300	94	9.75	3,481	8.87	1.1	0.16
	>300	109	11.23	5,855	14.92	0.75	0.1

**Table 3:** Training pattern recognition expression.

Serial number	The input layer						The output layer
	Elevation	Slope	Slope direction	Lithology	Distance to fault lines	Distance to drainage network	
1	0.37	0.21	0.11	0.17	0.1	0.16	0
2	0.37	0.21	0.14	0.18	0.25	0.15	0
3	0.37	0.21	0.11	0.18	0.22	0.15	0
4	0.37	0.25	0.1	0.18	0.22	0.15	0
5	0.59	0.27	0.1	0.12	0.25	0.16	1
6	0.59	0.21	0.14	0.12	0.22	0.15	0
7	0.59	0.25	0.14	0.17	0.21	0.15	0
8	0.59	0.25	0.13	0.17	0.1	0.15	0
9	0.37	0.21	0.14	0.17	0.1	0.15	0
10	0.37	0.25	0.13	0.17	0.1	0.15	0

Output layer: 0—no landslide and 1—landslide occurred.

**Table 4:** Predicting pattern recognition expression results.

Serial number	The input layer						Predicted value
	Elevation	Slope	Slope direction	Lithology	Distance to fault lines	Distance to drainage network	
1	0.37	0.21	0.1	0.08	0.1	0.1	0.339177359
2	0.37	0.21	0.1	0.08	0.1	0.16	0.363726631
3	0.37	0.21	0.1	0.08	0.1	0.15	0.33955281
4	0.04	0.27	0.11	0.08	0.1	0.15	0.111640847
5	0.37	0.16	0.12	0.08	0.1	0.14	0.340385979
6	0.04	0.21	0.13	0.08	0.1	0.1	0.115212578
7	0.04	0.27	0.11	0.08	0.1	0.1	0.113106344
8	0.04	0.16	0.11	0.08	0.1	0.15	0.114075971
9	0.37	0.16	0.11	0.08	0.1	0.14	0.339466781
10	0.37	0.21	0.1	0.08	0.1	0.16	0.333726631

## Data Availability

All datasets generated for this study have been included in the article.
